# Lipoprotein Glycosylation by Protein-*O*-Mannosyltransferase (MAB_1122c) Contributes to Low Cell Envelope Permeability and Antibiotic Resistance of *Mycobacterium abscessus*

**DOI:** 10.3389/fmicb.2017.02123

**Published:** 2017-11-02

**Authors:** Katja Becker, Klara Haldimann, Petra Selchow, Lukas M. Reinau, Michael Dal Molin, Peter Sander

**Affiliations:** ^1^Institute of Medical Microbiology, University of Zurich, Zürich, Switzerland; ^2^National Center for Mycobacteria, Zürich, Switzerland

**Keywords:** lipoprotein, glycosylation, *Mycobacterium abscessus*, post-translational modification, virulence, drug susceptibility

## Abstract

Lipoproteins are important components of the mycobacterial cell envelope due to their function in cell wall homeostasis and bacterial virulence. They are post-translationally modified with lipid- and glycosyl-residues in various species and interference with acylation or glycosylation leads to reduced growth and attenuated virulence in *Mycobacterium tuberculosis*. Lipoproteins are also expressed in the emerging and highly drug resistant pathogen *Mycobacterium abscessus* which frequently affects the lungs of patients with chronic pulmonary disease or cystic fibrosis. We investigated post-translational modification, acylation and glycosylation, of heterologously expressed (*M. tuberculosis* LppX and Mpt83) and endogenous (SodC) lipoproteins at the molecular level in *M. abscessus* and identified MAB_1122c as protein *O*-mannosyltransferase (Pmt). Both, heterologous and endogenous lipoproteins carried a characteristic lipid anchor with palmitic acid (C16), palmitoleic acid (C16:1), oleic acid (C18), or tuberculostearic acid (C19) modifications. Multiple hexose-moieties were detected in the *N*-terminal region of the model lipoproteins expressed in *M. abscessus*. Conservation of lipoprotein glycosylation in *M. tuberculosis* and *M. abscessus* was revealed and points toward the existence of an *O*-glycosylation motif or other regulatory mechanisms regarding this post-translational modification. Deletion of MAB_1122c prevented glycosylation and affected susceptibility to specific antibiotics which are large or target peptidoglycan synthesis and to lysozyme. Cell envelope permeability of *M. abscessus* Δ*pmt* was increased and mutant bacteria showed reduced survival inside macrophages. The results provide a link between post-translational modification of lipoproteins and the permeability of the mycobacterial cell envelope which stresses the importance of lipoproteins as components of this complex structure.

## Introduction

The cell envelope of mycobacteria is comprised of a plasma membrane, the periplasm and a lipid-rich mycobacterial outer membrane which contains arabinogalactan and mycolic acids. This thick and low-permeable structure contributes to persistence and high intrinsic drug resistance of mycobacteria (Brennan and Nikaido, 1995). Lipoproteins are important components of the mycobacterial cell envelope and in mycobacterial physiology since they fulfill various functions in membrane homeostasis, transmembrane signaling and adhesion to host cells (Sutcliffe and Harrington, 2004; Becker and Sander, 2016). In *Mycobacterium tuberculosis*, approximately 100 putative lipoproteins are expressed. They have been identified as immunogenic antigens (for review see Becker and Sander, 2016) and virulence factors since interruption of the lipoprotein synthesis pathway led to increased killing of bacilli inside macrophages and improved survival of infected mice (Sander et al., 2004; Rampini et al., 2008). LpqW for instance plays a role in synthesis of crucial lipoglycans, the lipoarabinomannans (Rainczuk et al., 2012) and RpfB (resuscitation promoting factor B) is involved in cleavage of peptidoglycan during resuscitation (Russell-Goldman et al., 2008). LppX mediates the transport of complex lipids, the phthiocerol-dimycocerosates, to the outer membrane where these serve as virulence factors (Sulzenbacher et al., 2006). Mpt83 is described as an adhesion factor (Carr et al., 2003). Lipoproteins are synthesized from pre-pro-lipoproteins through the consecutive action of diacylglyceryltransferase (Lgt), pro-lipoprotein signal peptidase (LspA) and *N*-acyltransferase (Lnt) (Tschumi et al., 2009, 2012; Brulle et al., 2013). These membrane-bound enzymes assemble the *N*-terminal lipid anchor, comprised of a diacylglycerol- and an *N*-acyl moiety, onto the conserved cysteine in the characteristic lipobox (LVI/ASTVI/GAS/C) (Kovacs-Simon et al., 2011).

Next to their three acyl moieties, some mycobacterial lipoproteins also carry glycosyl-residues. Protein glycosylation in *M. tuberculosis* is mediated by two enzymes, polyprenol-monophosphomannose-synthase (Ppm1) and protein-*O*-mannosyltransferase (Pmt). A mannose residue is transferred from the donor GDP-mannose to a polyprenol phosphate carrier in the plasma membrane by Ppm1. The mannosylated lipid carrier flips across the membrane so that the hexose is exposed at the periplasmic site. There, Pmt catalyzes the transfer of the mannose residue to a serine or threonine in the polypeptide chain (VanderVen et al., 2005). In mycobacteria to date only *O*-linked mannosylation has been described and the modification has been shown to be Sec-export dependent (Michell et al., 2003; VanderVen et al., 2005). Pmt might also be responsible for glycosylation of non-lipoproteins but it is clearly dispensable for synthesis of other crucial cell wall components like lipomannans or lipoarabinomannans (Horn et al., 1999; Liu et al., 2013). Lipoproteins are considered particularly important substrates of Pmt with regard to cell envelope homeostasis. In this study we investigate the effects of protein glycosylation deficiency on the mycobacterial cell envelope and therefore we focus on the lipoprotein glycosylating function of Pmt. In *M. tuberculosis*, *Mycobacterium smegmatis* or the closely related *Corynebacterium glutamicum* the lipoproteins LppX, Mpt83, LprI, LpqH, LprG, and SodC have been found with glycosyl-residues (Michell et al., 2003; Sartain and Belisle, 2009; Mohiman et al., 2012; Sethi et al., 2016; Alonso et al., 2017; Parra et al., 2017). The emerging pathogen *Mycobacterium abscessus*, a highly drug resistant fast-growing mycobacterium which infects the lungs of patients with chronic pulmonary disease or cystic fibrosis, also localizes lipoproteins in its cell envelope (Ripoll et al., 2009; Nessar et al., 2012). The bacteria’s capability to post-translationally modify these proteins and the effect of lipoprotein modification on *M. abscessus* homeostasis and virulence have not been investigated yet.

Here, we elucidated lipoprotein modification in *M. abscessus* at the molecular level. We characterized lipidation and glycosylation of heterologously expressed *M. tuberculosis* and endogenous *M. abscessus* lipoproteins by mass-spectrometry and revealed conservation of *O*-glycosylation throughout fast- and slow-growing mycobacterial species. The observation points to a universal but to date unknown motif for this post-translational modification. We identified MAB_1122c as Pmt, the lipoprotein glycosylating enzyme in *M. abscessus*. The role of lipoprotein glycosylation in *M. abscessus* cell envelope homeostasis, antibiotic resistance, and virulence in an *in vitro* model was assessed using a MAB_1122c deletion mutant. We show that lipoprotein glycosylation deficiency increases permeability of the *M. abscessus* cell envelope and alters susceptibility to large and bulky compounds and to antibiotics acting on the peptidoglycan layer. Furthermore, intracellular survival of glycosylation deficient *M. abscessus* is compromised.

## Materials and Methods

### Growth of Bacterial Strains

*Mycobacterium abscessus* was grown at 37°C with daily manual shaking in LB or Middlebrook 7H9-OADC-Tween supplemented with apramycin (apra, 50 μg/ml), kanamycin (kan, 50 μg/ml), or isoniazid (32 μg/ml) if required. Growth was monitored by measuring OD_600_ and bacteria were transformed by electroporation at 2.5 kV, 25 μF, 1000 Ω as previously described (Rominski et al., 2017a). Genomic DNA was isolated as described by Sander et al. (1995). For drug susceptibility assays cation-adjusted Müller-Hinton (CAMH) medium was used.

### Generation of *M. abscessus* ΔMAB_1122c

For deletion of the putative protein-*O*-mannosyltransferase MAB_1122c, 1.5 kb upstream and downstream fragments of MAB_1122c were amplified from *M. abscessus* genomic DNA using primers 1122c_up_F/1122c_up_R and 1122c_down_F/1122c_down_R (see Supplementary Table S1 for list of primers). Amplified fragments were cloned stepwise into the pSE-apra-KatG vector (Rominski et al., 2017c) using PscI/KpnI and KpnI/XbaI linkers for the up and down fragment, respectively. After transformation of the pSE-MAB1122c-KO vector into *M. abscessus*, selection with apramycin (positive) and isoniazid (negative) led to the unmarked deletion of MAB_1122c. Southern Blot analyses with probes I and II that were amplified from *M. abscessus* genomic DNA using primers PI_1122c_F/PI_1122c_R and PII_1122c_F/PII_1122c_R, respectively, verified recombination. For complementation, the MAB_1122c gene including its native promotor region was amplified from *M. abscessus* genomic DNA with primers C_1122c_F and C_1122c_R and inserted into the pMV361-kan vector via KpnI thereby generating pMV361-MAB1122c. Integration of the complementation vector in the *M. abscessus* Δ*pmt* genome was confirmed by Southern Blot.

### Lipoprotein Expression

Genes encoding LppX (Rv2945c; 702 bp) and Mpt83 (Rv2873; 663 bp) were amplified from *M. tuberculosis* genomic DNA with primers which introduce *C*-terminal, thrombin cleavable His- and HA- tags and allow fusion to selected promoters (LppX_F/LppX_R; Mpt83_F/Mpt83_R) (Supplementary Table S1). The 19 kDa and the pfbpB promoter were amplified from *M. tuberculosis* genomic DNA with primers 19 kDa_F/19 kDa_R and pfbpB_F/pfbpB_R. A fusion PCR with primers 19 kDa_F/LppX_R as well as pfbpB_F/Mpt83_R was performed to link the LppX and Mpt83 genes to the 19 kDa and pfbpB promoter, respectively. The fused pfbpB-Mpt83 sequence was cloned into the polyG-apra vector via HindIII and PstI. The fused 19 kDa-LppX sequence was first integrated into the pMV261-gentamicin vector then excised for insertion into the polyG-apra vector with HindIII and PstI. Gibson cloning (Bordat et al., 2015) was applied to insert endogenous *M. abscessus* SodC (MAB_4184c) into the polyG-apra vector. The gene (716 bp) was amplified from *M. abscessus* genomic DNA with primers SodC_F and SodC_R, introducing a Thrombin-cleavable His-and HA-Tag as well. The polyG-apra vector including the pfbpB promoter was amplified in two separate fragments from polyG-pfbpB-Mpt83 with primers polyG_a_F/polyG_a_R and polyG_b_F/polyG_b_R. Assembly was performed as described by Bordat et al. (2015).

Upon transformation of polyG-apra-LppX, polyG-apra-Mpt83, and polyG-apra-SodC into *M. abscessus* strains (see list of all strains used in this study in Supplementary Table S2), clones were selected on LB plates supplemented with apramycin and tested for protein expression by Western Blot. In brief, cells were harvested from liquid culture and disrupted with acid washed glass beads (≤106 μm, Sigma-Aldrich, Switzerland) in the FastPep system (4x 1 min at 6 m/s; MP Bio, United States). The lysate was loaded on a 12.5% Tris-HCl SDS Page gel (Criterion, BioRad, Switzerland) and proteins were detected with primary mouse anti-HA (1.300, Roche, Switzerland) and secondary goat anti-mouse-HRP (1:20,000, LabForce, Switzerland) antibodies.

### Protein Purification

*Mycobacterium abscessus* strains constitutively expressing LppX, Mpt83, or SodC were harvested and disrupted by FastPrep. Lysates were incubated with 2% *N*-Lauroylsarcosine sodium salt (SLS; Sigma-Aldrich, Switzerland) for 1 h at room temperature and kept at 4°C overnight. Ultracentrifugation for 1 h at 100,000 × *g* was applied to separate membrane fragments and unbroken cells from the solubilized lipoproteins. The lipoprotein containing supernatant after ultracentrifugation was diluted with dilution buffer (20 mM NaH_2_PO_4_, 0.5 M NaCl, 0.2% SLS, pH = 7.4) in a 1:1 ratio and loaded onto a Ni^2+^-column (His-Trap, GE Healthcare; Germany). Proteins were gradually eluted with elution buffer (20 mM NaH_2_PO_4_, 0.5 M NaCl, 0.2% SLS, 0.5 M imidazole, pH = 7.4) by fast-protein-liquid-chromatography (FPLC) using the ÄKTA Prime System (Amersham Bioscience, United Kingdom).

### Mass-Spectrometry

#### MALDI-TOF

Lipoprotein containing fractions after FPLC were precipitated with trichloroacetic acid (TCA) and separated on a 12.5% Tris-HCl SDS Page gel (Criterion, BioRad, Switzerland). Lipoprotein bands visualized with 0.3 M copper-chloride staining were excised and in-gel protein digestion with AspN was performed. Peptides were treated according to the Ujihara method as previously described (Ujihara et al., 2008) and samples were analyzed on an Ultraflex II MALDI-TOF/TOF instrument (Bruker, Germany) as noted by Tschumi et al. (2009). A mass tolerance of m/z≈1 was applied for the selection of modified peptide and fragment ions.

#### LC-ESI-MS

Pure fractions of lipoproteins were precipitated with TCA and resuspended in NH_4_HCO_3_ buffer (pH = 8.3) supplemented with 15% methanol and 1% dithiothreitol (DTT). Disulfide bonds were reduced with DTT at 50°C for 30 min and cysteines were alkylated by addition of 20 mM iodoacetamide at room temperature in the dark. Lipoproteins were digested with trypsin or AspN overnight at 37°C. Sample clean-up was performed with C_18_ ZipTips (Merck, Germany). Glycosylated peptide ions were identified by LC-ESI-MS/MS analysis with an Orbitrap Fusion mass-spectrometer (Thermo Fisher, Germany). Global settings included the method duration of 110 min and a spray voltage of 2000–2300 V for the positive ion and 600 V for the negative ion. MS-data were detected in the Orbitrap at a resolution of 120 k. The scan range was set to m/z = 400–2000 and the maximum injection time was 50 ms. The AGC Target value was 400,000. Peptide ions with a charge state of 2–7 were included and dynamic exclusion for 5 s after the first measurement was applied. A mass tolerance of 10 ppm was allowed. The minimum and maximum intensity thresholds were set to 50,000 and 1 × 10^20^, respectively. MS/MS-data were obtained upon higher-energy collision dissociation (HCD) fragmentation. Collision Energy was set to 30% and fragment ions were detected in the Orbitrap at a resolution of 120 k as well. The maximum injection time for MS/MS was 250 ms. The AGC Target value was set to 200,000 and one microscan was performed. Results were interpreted with Mascot using a database of mycobacterial protein sequences and allowing two missed cleavages and a mass tolerance of 0.04 Da for MS/MS-data. Carbamidomethyl (C) as a fixed modification and Gln →pyro-Glu (*N*-term Q), Oxidation (M), Hex-CID/HCD (S), and Hex-CID/HCD (T) as variable modifications were selected. Identified glycopeptides were confirmed by manual analyses of spectra. Detailed Mascot results of the analyses can be found in Supplementary Table S3.

### Minimal Inhibitory Concentration (MIC) Assay

Minimal inhibitory concentration (MIC) determination was performed according to the guidelines of the Clinical and Laboratory Standards Institute with the exception that incubation was at 37°C rather than at 30°C and as previously described (Rominski et al., 2017a). A fourfold difference in MIC values was considered significant. Ethambutol, isoniazid, bacitracin, vancomycin, rifampicin, and amikacin were dissolved in water and stored at -20°C until usage. Cefoxitin, ceftriaxone, penicillin G, and ampicillin were dissolved in water, aliquoted and stored as described by Rominski et al. (2017b). For the checkerboard MIC assay, twofold microdilutions of lysozyme (0.016–2 mg/ml) combined with bacitracin (0.5–64 mg/l) or vancomycin (0.008–1 mg/l) were produced in a 96-well plate format by an automated platform (Tecan Freedom EVO 100, Switzerland). Inocula were determined for all experiments at day 5 and experiments were validated when 4 × 10^5^ to 1.3 × 10^6^ CFUs/ml were counted. All MIC values were determined in three independent experiments after 5 days of growth at 37°C and the median of the measurements is depicted.

### Permeability Assay

*Mycobacterium abscessus* WT, Δ*pmt* and Δ*pmt::pmt* cultures in exponential growth phase were harvested, diluted to OD_600 nm_ = 0.5 in uptake buffer (50 mM KH_2_PO_4_, 5 mM MgSO_4_, pH = 7) and re-energized with 25 mM glucose. In a 96-well plate format, basic fluorescence (emission: 530 nm/extinction: 590 nm) was measured at t_-2 min_. 20 μM ethidium bromide was added to each sample subsequently. 0.1 mM Carbonyl-Cyanide 3-Chlorophenylhydrazone (CCCP) was used to control for the role of efflux pumps in ethidium bromide mediated fluorescence. Measurements were performed in triplicates and kinetics of ethidium bromide uptake were monitored from t_0 min_ on for 40 min at room temperature in a microplate reader (Bio Tek Synergy HT, Switzerland). Results were normalized against auto fluorescence of ethidium bromide and are depicted relative to the highest fluorescence measured. Statistical analysis of significance was performed by one-way ANOVA and Tukey’s Multiple Comparison Test (*P* < 0.05).

### Intracellular Survival Assay

Intracellular survival of *M. abscessus* strains in THP-1 macrophages was investigated as previously described (Roux et al., 2016). Macrophages were infected with bacteria at an MOI in the range of 0.2–2 for 2 h at 37°C. Extracellular bacteria were removed by extensive washes with PBS followed by incubation of cells with RPMI medium containing 250 μg/ml amikacin. After 1 h, amikacin concentration in the medium was reduced to 50 μg/ml for the remaining experiment. Statistical analysis of the results was performed by one-way ANOVA and Tukey’s Multiple Comparison Test (*P* < 0.001).

## Results

### Lipoprotein Acylation in *M. abscessus*

**FIGURE 1:**
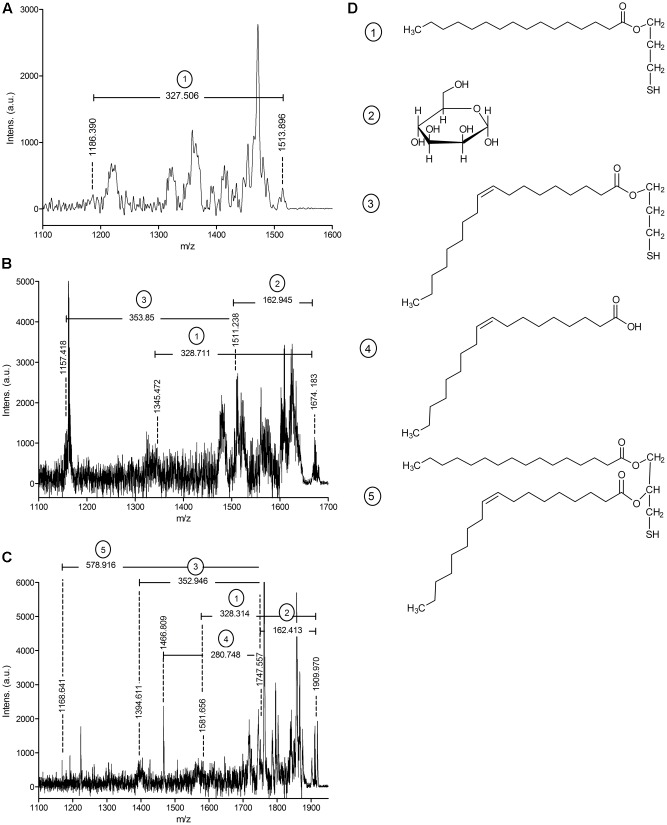
**MALDI-TOF/TOF analysis of Mpt83 (Rv2873) acylation in *Mycobacterium abscessus* WT.** **(A)** MS/MS spectrum of the *N*-terminal peptide ion of Mpt83 (CSSTKPVSQ) with palmitic acid/oleic acid (C16/C18) diacylglycerol modification (calculated m/z = 1513.355). Elimination of a palmitic acid-thiol-glyceryl (calculated m/z = 327.56) was observed. **(B)** MS/MS spectrum of the *N*-terminal peptide ion of Mpt83 with palmitic acid/ oleic acid (C16/C18) diacylglycerol + one hexose modification (calculated m/z = 1674.6). Elimination of a palmitic acid-thiol-glyceryl (calculated m/z = 327.56), the hexose (calculated m/z = 162.05) and oleic acid-thiol-glyceryl (calculated m/z = 353.66) was observed. **(C)** MS/MS spectrum of the *N*-terminal peptide ion of Mpt83 with palmitic acid/oleic acid (C16/C18) diacylglycerol + palmitic acid (C16) *N*-acyl + one hexose modification (calculated m/z = 1910.0). Elimination of palmitic acid-thiol-glyceryl (calculated m/z = 327.56), the hexose (calculated m/z = 162.05), oleic acid-thiol-glyceryl (calculated m/z = 353.66), oleic acid (calculated m/z = 281.5) and the palmitic acid/ oleic acid diacylglycerol (calculated m/z = 578.0) was observed. **(D)** Chemical structures of the eliminated fragments are depicted: (1) palmitic acid-thiol-glyceryl, (2) hexose, (3) oleic acid-thiol-glyceryl, (4) oleic acid, and (5) palmitic acid/oleic acid diacylglycerol.

*Mycobacterium tuberculosis* LppX (Rv2945c) and Mpt83 (Rv2873) were chosen as model lipoproteins for the analysis of acylation and glycosylation in *M. abscessus*. These proteins are stable, not toxic, and known to carry both modifications in other mycobacteria (Michell et al., 2003; Tschumi et al., 2009, 2012; Mohiman et al., 2012; Brulle et al., 2013). We also investigated SodC (MAB_4184c) as an endogenous *M. abscessus* protein which shows 61% homology to the lipidated and glycosylated *M. tuberculosis* protein (Rv0432) (Sartain and Belisle, 2009). Lipoprotein expression vectors polyG-apra-LppX, polyG-apra-Mpt83 and polyG-apra-SodC were transformed into *M. abscessus* for the expression of full-length His- and HA-tagged proteins. These were isolated by FPLC and analyzed by mass-spectrometry. The *N*-terminal peptide of heterologously expressed Mpt83 (AspN-digestion fragment: CSSTKPVSQ) was modified with palmitic acid/oleic acid (C16/C18) diacylglycerol as indicated by the detected peptide ion at m/z = 1513.899 (**Figure 1A**). This peptide ion was also identified with one additional hexose at m/z = 1674.183 (**Figure 1B**). A palmitic acid (C16) *N*-acyl modification in addition to the diacylglycerol and the hexose was found on the peptide ion at m/z = 1909.970 (**Figure 1C**). Fragmentation of these peptide ions in the MALDI-TOF/TOF analysis revealed the elimination of various fragments which confirmed the modifications (**Figure 1D**). Also LppX and SodC were shown to carry the characteristic lipid anchor of lipoproteins. Heterologously expressed LppX was modified with palmitic acid/oleic acid (C16/C18) diacylglycerol and a palmitic acid (C16) *N*-acyl residue in *M. abscessus*. Indication for a tuberculostearic acid/tuberculostearic acid (C19/C19) diacylglycerol modification at the *N*-terminal peptide of LppX (AspN-digestion fragment: CSSPKP) was obtained as well (**Table 1**). The *N*-terminal peptide of SodC (AspN-digestion fragment: CSP) was detected with a palmitoleic acid/oleic acid (C16:1/C18) diacylglycerol modification and one additional hexose (**Table 1**). The enzymatic machinery to synthesize tri-acylated lipoproteins at the conserved cysteine in their lipobox is thus present in *M. abscessus. In silico* homology searches with *M. tuberculosis* Lgt, LspA, and Lnt protein sequences identified MAB_2642c, MAB_2700c, and MAB_2206 as *M. abscessus* homologs.

**Table 1:** Lipidation of heterologous and native lipoproteins in *M. abscessus.*

		MS			MS/MS	
				
Protein	Peptide modification	Calculated m/z	Observed m/z	Eliminated fragment	Calculated m/z	Observed m/z
LppX (Rv2945c)	CSSPKP + Diacylglycerol (C16/C18)	1194.812	1195.202	C16/C18 diacylglyceryl	616.82	616.287
				C16/C18-thio-gIyceryI	585.72	586.134
	CSSPKP + Diacylglycerol (C16/C18) + *N*-acyl (C16)	1595.741	1595.843	1 Hex	1433.691	1434.299
				C18-thio-glyceryl ester + 1 Hex	1080.031	1080.294
	CSSPKP + Diacylglycerol (C19/C19)	1252.805	1253.342	C19/C19 diacylglycerol	616.705	616.096

SodC (MAB_4184c)	CSP + Diacylglycerol (C16:1/C18)	881.274	881.022	C16:1/C18 diacylglyceryl	305.274	305.084
				C18-thio-glyceryl ester	527.614	527.614
	CSP + Diacylglycerol (C16:1/C18) +1 Hex	1043.611	1043.263	C16:1/C18 diacylglyceryl +1 Hex	305.274	305.100
				C16:1-thio-glyceryl ester	718.051	717.495


### MAB_1122c Is a Putative Protein-*O*-Mannosyltransferase in *M. abscessus*

**FIGURE 2:**
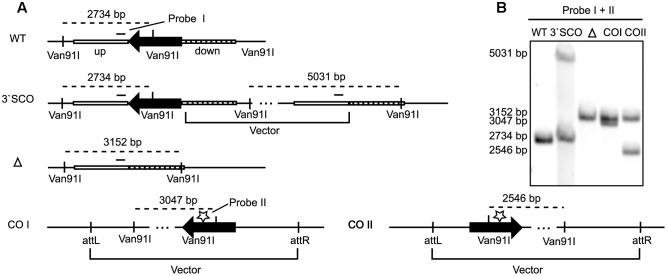
**MAB_1122c deletion in *M. abscessus*.** **(A)** Upon transformation of the allelic replacement vector pSE-MAB1122c-KO vector and positive selection (apramycin), homologous recombination in the down-fragment created a 3′ single crossover (3′ SCO). Subsequent second homologous recombination and negative selection (isoniazid) led to an unmarked knockout (Δ). Complementation was achieved through integration of the pMV361-MAB1122c vector (cw and acw orientation) at the *attP* site. A Van91I digest and two probes, in the up-fragment (probe I) and in the MAB_1122c gene (probe II), were used to verify each genotype by Southern Blot. **(B)** Southern Blot analysis of genomic DNA from MAB WT, 3′ SCO, MAB_1122c knockout (Δ) and the complemented strains (COI and COII) confirmed the genotypes. Probe I and II generated identical bands for *M. abscessus* WT (2734 bp) and 3′ SCO (Probe I: 2734 bp/5031 bp; Probe II: 2734 bp). The MAB_1122c deletion (Δ) hybridizes with probe I (3152 bp) while probe II detects the complemented genotypes (COI: 3047; COII: 2546).

In order to identify the gene responsible for the second post-translational modification on lipoproteins, we searched for homologs of mycobacterial lipoprotein glycosylating enzymes in *M. abscessus*. We performed an *in silico* search with *M. tuberculosis* (Rv1002) and *M. smegmatis* (Msmeg_5447) Pmt in *M. abscessus* ATTCC 19977 and identified MAB_1122c as putative Pmt with 68% homology to the *M. tuberculosis* and the *M. smegmatis* protein, respectively. In order to confirm gene annotation and to assess the role of lipoprotein glycosylation in the bacteria’s physiology we generated an unmarked MAB_1122c deletion mutant by transformation of *M. abscessus* with the allelic replacement vector pSE-MAB1122c-KO (**Figure 2A**). Single crossover transformants were selected on agar plates containing apramycin and identified by Southern Blot analysis (**Figures 2A,B**). Transformants were subsequently subjected to counter selection in the presence of isoniazid and also analyzed by Southern Blot (**Figures 2A,B**). The *M. abscessus* Δ*pmt* mutant was transformed with the complementation vector pMV361-MAB1122c. Genomic DNA was used to confirm integration of the vector by Southern Blot (**Figures 2A,B**).

### Protein-*O*-Mannosyltransferase (MAB_1122c) Glycosylates Lipoproteins in *M. abscessus*

**FIGURE 3:**
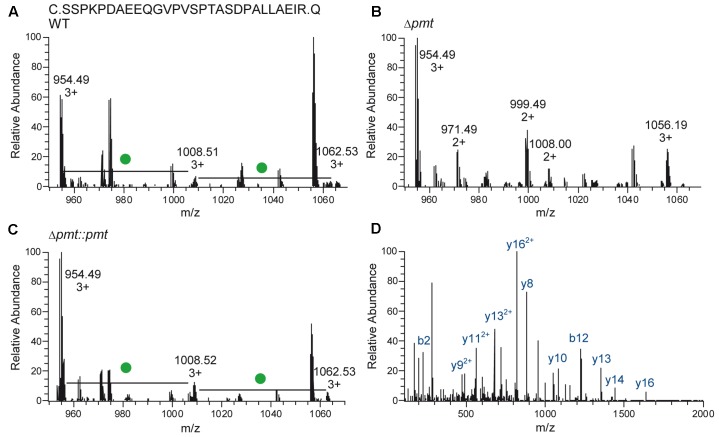
**Glycosylation of LppX (Rv2945c) in *M. abscessus*.** **(A–C)** Mass-spectra representative for LC-ESI-MS analyses of LppX purified from *M. abscessus* WT, Δ*pmt* and Δ*pmt::pmt* are shown. The *N*-terminal, tryptic peptide ion (SSPKPDAEEQGVPVSPTASDPALLAEIR) of LppX was detected unmodified (calculated m/z = 954.489, 3+), with one hexose (calculated m/z = 1008.505, 3+) and with two hexoses (calculated m/z = 1062.522, 3+) in *M. abscessus* WT **(A)** and *M. abscessus* Δ*pmt::pmt* **(C)**. Differences of m/z = 162.05/3 = 54 between the peptide ion peaks reflect hexose modification (green dot). In *M. abscessus* Δ*pmt* only the unmodified peptide ion (calculated m/z = 954.489, 3+) was detected **(B)**. **(D)** The MS/MS spectrum of the unmodified peptide ion found in *M. abscessus* WT (depicted) is also representative for the *N*-terminal peptide ions of LppX from *M. abscessus* Δ*pmt* and Δ*pmt::pmt* since glycosylation is eliminated during higher-energy collision dissociation (HCD) fragmentation. Detection of predicted fragment ions (y- and b-ions; Supplementary Table S3) confirms the identity of modified and unmodified peptide ions.

Heterologous expression of LppX (Rv2945c) and Mpt83 (Rv2873) in *M. abscessus* WT, Δ*pmt* and Δ*pmt::pmt* and subsequent LC-ESI-MS analysis revealed that in *M. abscessus* WT the lipoprotein LppX is glycosylated with one to two hexoses (**Figure 3A**). Mpt83 was more extensively glycosylated with up to six hexoses (**Table 2**). Glycosylated peptide ions eluted earlier than non-glycosylated peptide ions from the liquid-chromatography column due to increased hydrophilicity [see retention time (RT); **Table 2**]. All hexoses, presumably mannoses as described for other mycobacteria (Dobos et al., 1996; Michell et al., 2003), were identified in the tryptic *N*-terminal peptides (LppX: SSPKPDAEEQGVPVSPTASDPALLAEIR/ Mpt83: PATSPAAPVTTAAMADPAADLIGR). Analysis of lipoprotein glycosylation in *M. abscessus* WT compared to *M. abscessus* Δ*pmt* and *M. abscessus* Δ*pmt::pmt* revealed that MAB_1122c encodes for Pmt and is essential for lipoprotein glycosylation in *M. abscessus.* LppX purified from *M. abscessus* Δ*pmt* was not glycosylated (**Figure 3B**) but glycosylation was restored in the complemented strain (**Figure 3C**). Mpt83 glycosylation was also abolished in *M. abscessus* Δ*pmt* but was fully restored in the complemented *M. abscessus* Δ*pmt::pmt* strain (**Table 2**). The identity of modified peptide ions was confirmed by manual analysis of corresponding MS/MS-spectra (**Figure 3D**). Due to the loss of glycosylation during HCD-fragmentation, the MS/MS-spectrum depicted in **Figure 3D** is representative both for the glycosylated and non-glycosylated *N*-terminal peptide ion of LppX (see Supplementary Table S4 for predicted fragment ions). In order to confirm that also endogenous *M. abscessus* proteins are glycosylated, we recombinantly expressed and subsequently analyzed *M. abscessus* SodC (MAB_4184c). LC-ESI-MS analysis indicated that this protein is glycosylated with up to nine hexoses in *M. abscessus* WT. Glycosylation sites were located in the *N*-terminal peptide (AspN-digestion fragment: DQQASTTPGTTPPVWTGSTAPATAGEHGGHGGGEQPVPAGEKLNATLKLA; **Table 3**). Identification of glycosylation of *M. abscessus* SodC indicated that also endogenous lipoproteins are glycosylated by Pmt (MAB_1122c) in *M. abscessus*.

**Table 2:** Glycosylation of Mpt83 (Rv2873) in *M. abscessus* WT, Δ*pmt* and Δ*pmt::pmt.*

		Mpt83 (Rv2873)								
		
		K.PATSPAAPVTTAAMADPAADLIGR.G + Oxidation (M)								
		
		WT			Δpmf			Δ*pmt::pmt*		
				
Glycosylation	Calculated m/z	Observed m/z	z	RT	Observed m/z	z	RT	Observed m/z	z	RT
No hexose	761.05	761.05	3+	46.3	761.05	3+	46.25	761.05	3+	46.2
1 Hexose	815.07	815.072	3+	44.69				815.07	3+	44.56
2 Hexoses	869.09	869.09	3+	42.18				869.08	3+	42.23
3 Hexoses	923.10	923.10	3+	41.32				923.1	3+	41.28
4 Hexoses	977.12	977.12	3+	40.9				977.12	3+	40.08
5 Hexoses	1031.14	1031.14	3+	39.53				1031.14	3+	39.47
6 Hexoses	1085.15	1085.15	3+	38.76				1085.15	3+	38.95


**Table 3:** Glycosylation of SodC (MAB_4148c) in *M. abscessus* WT.

		SodC (MAB_4184c)		
		
		P.DQQASTTPGTTPPVWTGSTAPATAGEHGGHGGGE QPVPAGEKLNATLKLA.D		
		
		WT		
		
Glycosylation	Calculated m/z	Observed m/z	z	RT
No hexose	970.67	970.485	5+	65.97
1 Hexose	1003.08	1002.895	5+	64.33
2 Hexoses	1035.49	1035.305	5+	63.32
3 Hexoses	19067.90	1067.715	5+	62.77
4 Hexoses	1100.32	1100.125	5+	61.06
5 Hexoses	1132.72	1132.535	5+	59.69
6 Hexoses	1165.13	1164.945	5+	59.02
7 Hexoses	1197.54	1197.357	5+	58.87
8 Hexoses	1229.95	1229.767	5+	57.96
9 Hexoses	1262.36	1262.175	5+	57.63


### *M. abscessus* Δ*pmt* Is Susceptible to Bulky Antibiotics and to Compounds Acting on Peptidoglycan

**FIGURE 4:**
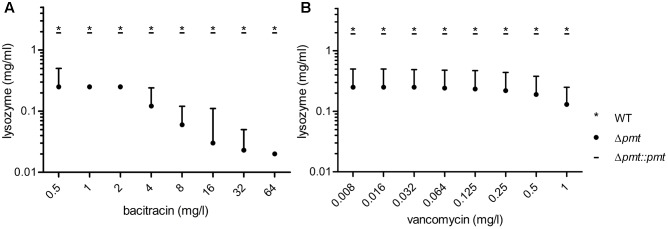
**Synergistic effects of bacitracin and vancomycin with lysozyme.** Antibiotic dilution series starting with concentrations fourfold below the MIC value for bacitracin **(A)** and vancomycin **(B)** determined for *M. abscessus* Δ*pmt* were combined with lysozyme concentrations of 0.016–2 mg/ml. Maximum tolerated concentrations of both compounds are depicted for *M. abscessus* WT (^∗^), Δ*pmt* (∙) and Δ*pmt::pmt* ( **_** ). Results of three independent experiments are represented and the median of tolerated concentrations with the range of all measurements is shown.

The effect of lipoprotein glycosylation deficiency on antibiotic susceptibility of *M. abscessus* was assessed in a MIC assay. *M. abscessus* Δ*pmt* showed increased susceptibility to all tested β-lactams (cefoxitin, ceftriaxone, penicillin G, and ampicillin) as well as to the high molecular weight antibiotics (Supplementary Figure S1) bacitracin (MIC = 256 mg/l), vancomycin (MIC = 4 mg/l), and rifampicin (MIC = 64 mg/l). Also susceptibility to lysozyme (MIC = 0.5 mg/ml) was elevated (**Table 4**). In contrast, susceptibility to smaller antibiotics interfering with the synthesis of other cell wall components (ethambutol, isoniazid) or affecting translation (amikacin) was not altered. Tolerance for the β-lactams, bacitracin, vancomycin, rifampicin, and lysozyme comparable to that seen in WT bacteria was restored in *M. abscessus* Δ*pmt::pmt*. In order to investigate potential synergistic effects between the bulky and peptidoglycan affecting antibiotics bacitracin/vancomycin and lysozyme we conducted a checkerboard titration assay. Specifically, we tested subinhibitory concentrations of these antibiotics in combination with the peptidoglycan cleaving enzyme. Highest antibiotic concentrations tested were fourfold below MIC values determined for *M. abscessus* Δ*pmt* (**Table 4**). The maximum tolerated concentrations of antibiotic and lysozyme are depicted in **Figure 4**. Susceptibility of *M. abscessus* WT and *M. abscessus* Δ*pmt::pmt* to the antibiotics and lysozyme was unaffected by the combination of these compounds (**Figures 4A,B**). However, *M. abscessus* Δ*pmt* became more susceptible to lysozyme with increasing concentrations of bacitracin. At the highest bacitracin concentration, which is still fourfold below the MIC value for *M. abscessus* Δ*pmt*, as little as 0.02 mg/ml lysozyme were tolerated (**Figure 4A**). This observation indicates synergistic effects of lysozyme and bacitracin against *M. abscessus* Δ*pmt*. Tolerated concentrations of lysozyme remained at 0.25 mg/ml until vancomycin concentrations rose to 0.5 and 1 mg/l (**Figure 4B**). Since only higher concentrations of vancomycin affected susceptibility of *M. abscessus* Δ*pmt* to lysozyme, vancomycin and lysozyme exhibit weaker synergistic effects.

**Table 4:** Antibiotic susceptibility of *M. abscessus* WT, Δ*pmt* and Δ*pmt::pmt.*

	Strain		
	
Compound (mg/l)	WT	Apmf	*Apmt::pmt*
Ethambutol	64	64	64
Isoniazid	512	1024	512
Bacitracin	>2048	256	>2048
Vancomycin	128	4	128
Rifampicin	256	64	256
Amikacin	2	2	2
Cefoxitin	64	16	32
Ceftriaxone	128	32	128
Penicillin	>512	256	>512
Ampicillin	>512	128	>512
Lysozyme	>2000	500	>2000


### Increased Cell Wall Permeability in *M. abscessus* Δ*pmt*

**FIGURE 5:**
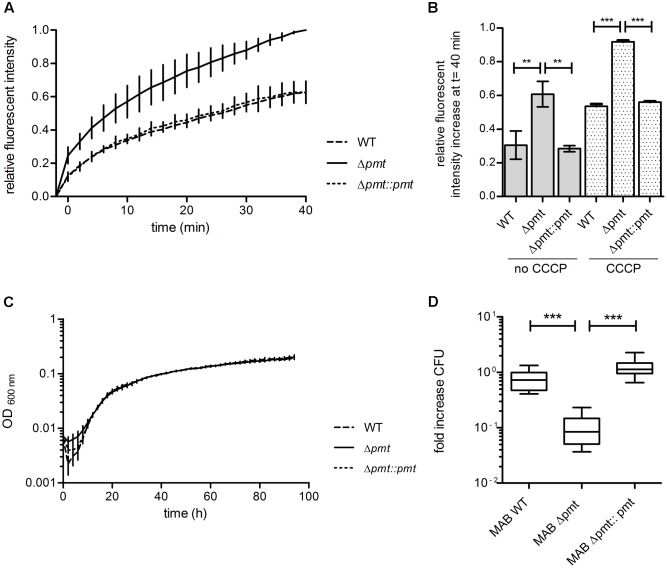
**Permeability and virulence of *M. abscessus* WT, Δ*pmt* and Δ*pmt::pmt*.** **(A)** Fluorescent intensity of *M. abscessus* cultures at t _-2 min_ and after subsequent addition of ethidium bromide was measured to detect uptake and incorporation of the fluorescent molecule into DNA. Kinetics of ethidium bromide uptake from t _0 min_ onward were measured for 40 min at room temperature. Results of three independent experiments are shown. Fluorescent intensities were normalized against autofluorescence of ethidium bromide in uptake buffer and are depicted relative to the maximal intensity measured. **(B)** The relative fluorescent intensity increase after 40 min was calculated for all strains with and without CCCP treatment and statistically analyzed by one-way ANOVA and Tukey’s Multiple Comparison Test (^∗∗^*P* < 0.05; ^∗∗∗^*P* < 0.001). Error bars reflect the standard deviation in three independent experiments. **(C)** Growth curve in Middlebrook 7H9-OADC-Tween at 37°C with regular shaking for *M. abscessus* WT, Δ*pmt* and Δ*pmt::pmt*. Results of three independent experiments with standard deviations are depicted. **(D)** Survival of *M. abscessus* WT, Δ*pmt* and Δ*pmt::pmt* in THP-1 macrophages 3 days after infection. Results of multiple independent experiments are shown (WT: *n* = 3; Δ*pmt*: *n* = 3; Δ*pmt::pmt*: *n* = 2). One-way ANOVA and Tukey’s Multiple Comparison Test statistical analysis were performed (^∗∗∗^*P* < 0.001).

Based on the susceptibility of *M. abscessus* Δ*pmt* to large antibiotics which affect peptidoglycan and the observed synergistic effects with lysozyme, we hypothesized that the permeability of the cell envelope of this strain might be increased due to structural effects of lipoprotein glycosylation deficiency. We therefore conducted an ethidium bromide uptake assay to test for altered permeability in the MAB_1122c deletion mutant. Relative fluorescent intensity measurements indicated that *M. abscessus* Δ*pmt* takes up ethidium bromide more efficiently than *M. abscessus* WT and *M. abscessus* Δ*pmt*::*pmt* (**Figure 5A**). This elevated level of ethidium bromide uptake in the mutant strain was also observed upon inhibition of efflux processes with 0.1 mM CCCP while overall fluorescent intensity in all strains was higher due to increased ethidium bromide accumulation. The total increase in fluorescent intensity after 40 min was significantly higher in *M. abscessus* Δ*pmt* as compared to WT and the complemented strain both with and without CCCP treatment (**Figure 5B**). Combined, these results point toward an increased permeability of the *M. abscessus* Δ*pmt* cell envelope.

### Glycosylation Deficiency Reduces Intracellular Survival of *M. abscessus* Δ*pmt*

*In vitro* growth of *M. abscessus* strains was assessed in Middlebrook 7H9-OADC-Tween medium at 37°C with regular shaking and no difference in growth rate was observed between *M. abscessus* WT, Δ*pmt* and Δ*pmt*::*pmt* (**Figure 5C**). In order to investigate potential effects of glycosylation deficiency on virulence of *M. abscessus* we conducted an intracellular survival assay with the human monocyte-macrophage THP-1 cell line. *M. abscessus* WT and *M. abscessus* Δ*pmt*::*pmt* survived inside the macrophages during a 3 days course of infection (**Figure 5D**). CFU counts for *M. abscessus* Δ*pmt* significantly decreased by 0.8 log within 3 days of infection as compared to day 0 (**Figure 5D**). This indicates killing of *M. abscessus* Δ*pmt* inside infected macrophages and represents virulence attenuation of the MAB_1122c deletion mutant as compared to *M. abscessus* WT and *M. abscessus* Δ*pmt::pmt.*

## Discussion

In this study we investigated post-translational modification of lipoproteins and the function of lipoprotein glycosylation in the emerging pathogen *M. abscessus*. We show that *M. abscessus* tri-acylates lipoproteins with fatty acids obtained from the phospholipids in its plasma membrane. The observed lipid modifications include palmitic acid (C16), oleic acid (C18), palmitoleic acid (C16:1), and tuberculostearic acid (C19). Consistently with observations in *M. smegmatis* and *M. bovis* BCG, the membrane anchor of lipoproteins in *M. abscessus* is comprised of a heterogeneous combination of fatty acids which can vary per protein-molecule (Brulle et al., 2013). We demonstrate that lipoproteins can be glycosylated with a variable number of hexoses in *M. abscessus*. This modification is mediated by MAB_1122c which encodes for Pmt and is essential for lipoprotein glycosylation in the emerging pathogen. Modification by Pmt marks the first step in lipoprotein glycosylation in which the mannose is attached to the exported protein (Liu et al., 2013). Consequently, lipoprotein glycosylation in *M. abscessus* Δ*pmt* is abolished completely. All glycosyl-residues in *M. abscessus* were detected in the *N*-terminal region of the model lipoproteins as described for lipoproteins expressed in other mycobacteria as well (Herrmann et al., 1996; Michell et al., 2003; Sartain and Belisle, 2009; Mohiman et al., 2012; Sethi et al., 2016; Parra et al., 2017). This observation indicates the existence of a conserved motif which is recognized by Pmt from various mycobacterial species and directs site-specific glycosylation of lipoproteins in fast-and slow-growing mycobacteria (Sartain and Belisle, 2009; Smith et al., 2014). Our notion, that heterologously expressed lipoproteins like Mpt83 carry glycosyl-residues in the same *N*-terminal region and probably at identical residues as when expressed in their native environment supports this hypothesis (Michell et al., 2003). Despite intensive investigations by Smith et al. (2014) the potential consensus sequence for *O*-glycosylation sites has to be further defined and verified in mycobacteria. Another possible explanation for the exclusive and conserved localization of glycosyl-moieties could be that only serine or threonine residues in the *N*-terminal region of lipoproteins are accessible for Pmt during Sec-export. The SodC protein was detected with up to nine mannoses in *M. tuberculosis* (Sartain and Belisle, 2009) and carried the same number of hexoses in *M. abscessus*. However, a mechanism for glycosyl-chain length control in mycobacteria is unknown to date. It is also not clear yet whether detected mannoses are attached to one amino acid each or whether oligosaccharide chains are formed in *M. abscessus*. Extensive glycosylation as seen for Mpt83 and SodC, however, suggests that at least short mannose chains occur. Elongation of these would be performed not by Pmt but by PimE (Rv1159), an enzyme also involved in the synthesis of other membrane components such as LMs and LAMs where it mediates the addition of α-(1,2) linked mannoses to PIM molecules (Liu et al., 2013). In *M. abscessus* MAB_1304 has 64% homology to *M. tuberculosis* PimE.

Next to the structural analysis of lipoprotein modification we show that glycosylation deficiency in *M. abscessus* alters bacterial susceptibility to large and bulky compounds and to drugs and enzymes which affect peptidoglycan. Due to their thick and almost impermeable outer membrane like structure and the expression of β-lactamases mycobacteria are usually not susceptible to antibiotics acting on the peptidoglycan layer (Brennan and Nikaido, 1995; Nessar et al., 2012). Nevertheless, we observed synergistic effects of both bacitracin and vancomycin with lysozyme in the Δ*pmt* strain. We confirmed by *in silico* analysis that *M. abscessus* does not express a homolog of the lysozyme inhibitor LprI which has been described in *M. tuberculosis* recently and could affect lysozyme MIC-testing (Sethi et al., 2016). Particularly the antimicrobial activity of the bacitracin-lysozyme combination was strong. The observed effects on antibiotic susceptibility and intracellular survival might occur due to structural alterations in the cellular envelope of *M. abscessus* Δ*pmt* upon lipoprotein glycosylation deficiency. This hypothesis is in line with the increased cell envelope permeability seen in the ethidium bromide uptake assay and could explain why particularly large antibiotics like bacitracin, vancomycin, and rifampicin (Supplementary Figure S1) can affect the mutant and why the β-lactams can better reach their target, the peptidoglycan layer. Also for the *M. tuberculosis* Rpf^null^ mutant deficient for all five mycobacterial Rpfs it was described that the permeability of the outer membrane like structure is increased which led to a change in susceptibility to various β-lactams (Wivagg et al., 2014). This observation supports the link between an altered cell wall homeostasis and increased susceptibility to a broad range of large and bulky as well as to peptidoglycan affecting compounds which we describe in this study for the *M. abscessus* Δ*pmt* mutant. The details of how lipoprotein glycosylation deficiency affects cell permeability and drug susceptibility of *M. abscessus*, however, remain elusive. One possibility is that specific lipoproteins lose function without their post-translational modification but to date there is no indication for a functional requirement of glycosylation on lipoproteins. For SodC it was shown that glycosylation deficiency affects localization of the lipoprotein but is dispensable for its function (Sartain and Belisle, 2009). Also for LprI in *M. tuberculosis* it was reported that glycosylation is crucial for correct localization of the protein (Sethi et al., 2016). It is therefore likely that the localization of specific lipoproteins is altered upon glycosylation deficiency. This could affect the cell envelope structure directly or interfere with the homeostasis through indirect alterations in membrane composition. In *M. tuberculosis*, lipoproteins such as LpqW (conversion of phosphoinositol mannosides to lipomannans and lipoarabinomannans) or LppX (transport of phthiocerol dimycocerosates) for instance are important players in the synthesis and transport of cell wall components, respectively (Sulzenbacher et al., 2006; Rainczuk et al., 2012). Homologs of LpqW (MAB_1315) and the LppX_LprAFG superfamily (MAB_2806) are encoded in *M. abscessus.* Incorrect localization of these or other lipoproteins in *M. abscessus* Δ*pmt* could affect cell wall homeostasis because their substrates might not be synthesized or located correctly.

Based on the obtained results it cannot be excluded that Pmt possess an additional function independent of lipoprotein glycosylation that leads to the observed phenotypes. The enzyme might be involved in the glycosylation of other (non-lipo-) proteins but does not play a role in lipomannan or lipoarabinomannan synthesis (Liu et al., 2013). Due to the specific localization of lipoproteins in the cell envelope, however, it is plausible that the observed phenotypes are assigned to a deficiency in lipoprotein glycosylation. Pmt deletion mutants of *M. smegmatis* and *M. tuberculosis* have been investigated (Liu et al., 2013). The fast-growing, non-pathogenic *M. smegmatis* Δ*pmt* strain showed WT-like growth and susceptibility to the antibiotic ciprofloxacin while exhibiting only slightly decreased tolerance to cell wall stress by SDS. Electron microscopy analysis of the cell surface did not reveal obvious structural alterations and intracellular survival of the mutant was not assessed. Interestingly, the slow-growing, pathogenic *M. tuberculosis* Δ*pmt* strain, however, was attenuated in *in vitro* growth and showed reduced virulence in immunocompromised mice (Liu et al., 2013). The observed phenotype was very recently attributed to increased lipoarabinomannan production in the Δ*pmt* strain and secretion of this molecule partly bound to LprG. This led to a stronger inflammatory response via TLR-2 activation and could be a rationale for the attenuated virulence of *M. tuberculosis* Δ*pmt* (Alonso et al., 2017) and thus potentially also of *M. abscessus* Δ*pmt*. However, in the fast-growing pathogenic *M. abscessus* we see an intermediate phenotype: attenuated survival in macrophages but no *in vitro* growth defect. It is tempting to speculate that the growth rate of the mycobacterial species could affect the severity of the glycosylation deficient phenotype and that lipoprotein glycosylation is more crucial in pathogenic mycobacteria but this requires further investigation.

It will be of interest to explore the detailed downstream effects of lipoprotein glycosylation deficiency on the cell envelope permeability of *M. abscessus* in order to elucidate how the observed phenotypes arise on a molecular level. Furthermore, it remains an open question how glycosylation is exclusively directed to the *N*-terminal region of lipoproteins in all mycobacterial species investigated so far and whether mannose chain length is regulated and conserved. The results of this study contribute to a more detailed understanding of the fundamental biology and the drug resistance of the emerging pathogen *M. abscessus*. They can be used as a foundation for further exploration of lipoproteins in the context of the complex mycobacterial cell envelope in non-tuberculous but pathogenic mycobacteria.

## Author Contributions

KB and PSa conceived and designed the study. KB, KH, PSe, and LR acquired and analyzed the data. KB drafted the manuscript and PSa critically revised it. All authors approved the final version of the manuscript.

## Conflict of Interest Statement

The authors declare that the research was conducted in the absence of any commercial or financial relationships that could be construed as a potential conflict of interest.
