# Supplementary material for: Lipoprotein Glycosylation by Protein-O-Mannosyltransferase (MAB_1122c) Contributes to Low Cell Envelope Permeability and Antibiotic Resistance of Mycobacterium abscessus
Source: Front Microbiol. 2017 Nov 2;8:2123. doi: 10.3389/fmicb.2017.02123 (PMC5673659; doi:10.3389/fmicb.2017.02123)
Supplement: Supplementary file 2 [file Data_Sheet_1.docx]

***Supplemental Material***

**Lipoprotein Glycosylation by Protein-*O*-Mannosyltransferase (MAB_1122c) Contributes to Low Cell Envelope Permeability and Antibiotic Resistance of *Mycobacterium abscessus***

**Supplementary Table S1: Primers used in this study**

**Supplementary Table S2: *M. abscessus* strains used in this study**

**Supplementary Table S3: Mascot analysis of LC-ESI-MS/MS data**

see Supplementary_Table_S3.xlsx file

**Supplementary Table S4: MS/MS- Fragment Ion table LppX**

based on Proteomics Toolkit: http://db.systemsbiology.net:8080/proteomicsToolkit/FragIonServlet.html

**
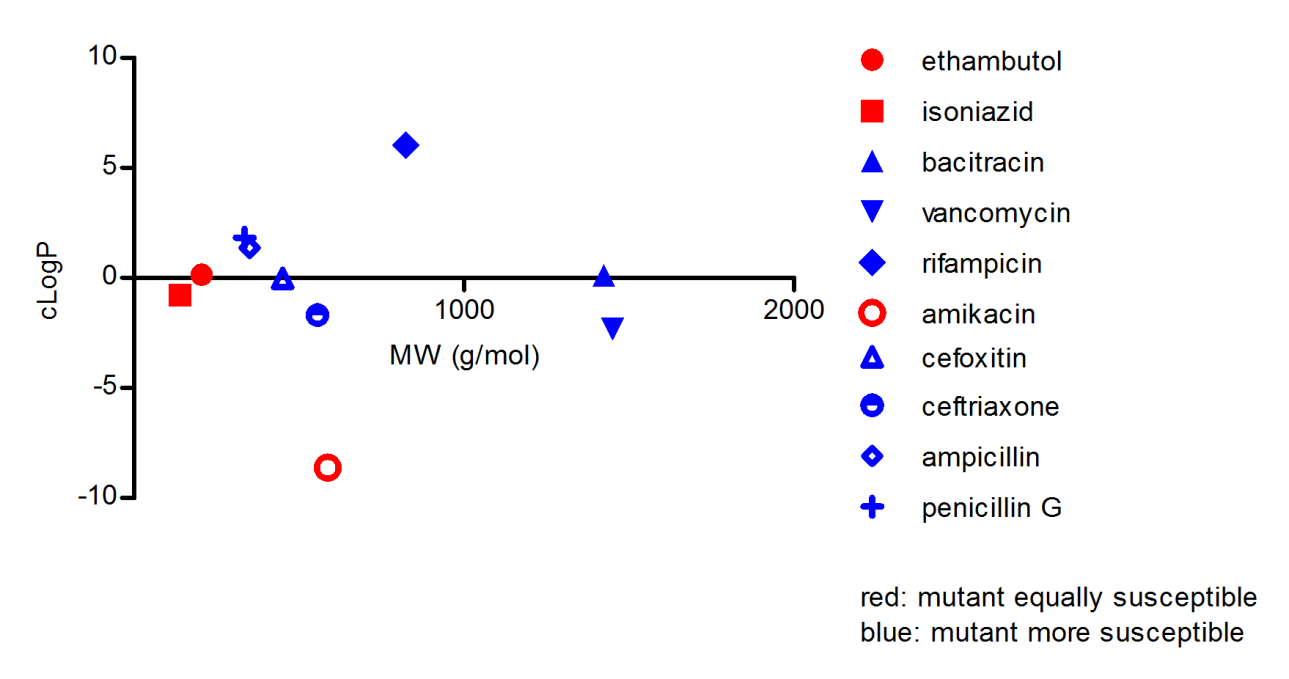
**

**Supplementary Figure S1:** Molecular characteristics of antibiotics tested in MIC assay. Hydrophobicity (cLogP) and molecular weight (MW) are depicted for each compound. Those compounds towards which susceptibility is affected in the *M. abscessus* Pmt deletion mutant are shown in blue.
